# Effect of smoking on the development and outcomes of inflammatory bowel disease in Taiwan: a hospital-based cohort study

**DOI:** 10.1038/s41598-022-11860-y

**Published:** 2022-05-10

**Authors:** Bor-Cheng Chen, Meng-Tzu Weng, Chin-Hao Chang, Ling-Yun Huang, Shu-Chen Wei

**Affiliations:** 1 School of Medicine, College of Medicine, National Taiwan University, Taipei, Taiwan; 2 Department of Medical Research, National Taiwan University Hospital Hsin-Chu Branch, HsinChu County, Taiwan; 3 Department of Medical Research, National Taiwan University Hospital, National Taiwan University, Taipei, Taiwan; 4 Clinical Trial Center, National Taiwan University Hospital, National Taiwan University, Taipei, Taiwan; 5 Division of Hepatology and Gastroenterology, Department of Internal Medicine, College of Medicine, National Taiwan University Hospital, National Taiwan University, No. 7 Chung-Shan South Road, Taipei, Taiwan

**Keywords:** Diseases, Gastroenterology

## Abstract

Smoking influences the risks of inflammatory bowel disease (IBD). A hospital-based cohort was conducted to evaluate the effect of smoking on the development and outcomes of IBD, with age, sex and comorbidities matched non-IBD controls from the National Health Interview Survey database of Taiwan. 700 IBD patients (360 ulcerative colitis (UC), 340 Crohn’s disease (CD)) were analyzed for outcomes; and 575 patients (297 UC, 278 CD) were analyzed for prevalence. Smoking prevalence was significantly lower in UC patients than controls (20.9% vs. 30.4%, *p* < 0.01), but no difference between CD patients and controls (19.8% vs. 22.1%, *p* = 0.60). UC smokers had fewer admissions (1.6 vs. 2.5, *p* < 0.05) but higher rates of new cancer development (16% vs. 6.7%, *p* < 0.05) and mortality (16% vs. 4.9%, *p* < 0.01) than nonsmokers. CD smokers tended to have higher rates of stricturing and penetrating diseases (*p* < 0.05), and higher surgery risk (60.3% vs. 38.3%, *p* < 0.01) than nonsmokers. Smoking prevents UC occurrence and is associated with fewer hospitalization but increases risks of cancer and mortality. By contrast, smoking does not affect CD occurrence but is related to more aggressive behavior which results in a higher surgical rate.

## Introduction

Inflammatory bowel disease (IBD) is a chronic inflammatory condition affecting the gastrointestinal tract. The two main IBD forms are ulcerative colitis (UC) and Crohn disease (CD). The definite IBD etiologies remain unknown. According to current studies, IBD etiologies are multifactorial, including genetic and environmental causes^1^. Smoking has been reported as one of the environmental factors related to IBD^1^. The World Health Organization (WHO) reported that > 1.1 billion people aged ≥ 15 years smoked tobacco in 2016. The age-standardized prevalence of tobacco smoking in 2016 for both sexes globally was 19.9%, with the lowest prevalence in Africa (9.8%), the highest prevalence in Europe (29.4%), and an intermediate prevalence of 16.9% in South East Asia^2^. Thomas et al.^3^ analyzed smoking prevalence in global IBD cohorts and showed that never-smokers in the newly diagnosed CD population in the West increased over the past two decades, particularly in the United Kingdom (+ 26.6%) and Sweden (+ 11.2%). By contrast, the proportion of never-smokers at diagnosis in the IBD population decreased in Asia, particularly in China.

The relationship between smoking and IBD was first described by Harries et al.^4^ who noted that patients with UC tended to be nonsmokers. Smoking is associated with increased CD risk and decreased UC risk in the Western population^4,5^. Mahid^5^ revealed an association between current smoking and CD (odds ratio [OR], 1.76; 95% confidence interval [CI], 1.40–2.22) and former smoking and UC (OR, 1.79; 95% CI, 1.37–2.34). Additionally, current smoking had a protective effect on UC development in comparison with controls (OR, 0.58; 95% CI, 0.45–0.75). In a prospective cohort study of 200,000 women^6^, the hazard ratio (HR) of CD was 1.90 (95% CI, 1.42–2.53) among current smokers compared with never-smokers. However, the HR of UC was 0.86 (95% CI, 0.61–1.20) among current smokers compared with never-smokers, indicating no evidence of increased UC risk in current smokers. Moreover, UC risk significantly increased within 2–5 years of smoking cessation (HR, 3.06; 95% CI, 2.00–4.67) and remained persistently high for 20 years. For disease behavior, Louis^7^ reported that active smoking was associated with the development of stricturing and penetrating CD, particularly perianal penetrating disease, whereas other studies^8,9^ have shown that disease behavior changes over time and is independent of smoking.

The association between smoking and IBD has not been well studied and is controversial in the Asian population. Takahashi^10^ reported a bimodal distribution of UC onset age in Japan, which is similar to that in Western countries. In addition, in recent years, smoking cessation may partly contribute to an increase in late-onset UC in Japan. Wang^11^ conducted a multicenter case–control study in patients of China, India, and the United States and reported that UC patients were more likely to be former smokers than controls in China, but both UC and CD patients had smoking status similar to controls at diagnosis in India. Zhai^12^ conducted a single-center retrospective study in China and reported that heavy smokers had lower UC severity than other smokers and nonsmokers based on the Modified Mayo score, and that current smokers needed fewer corticosteroids than nonsmokers. Other than these studies, few studies have focused on the effect of smoking on IBD in Asia. Moreover, no findings are available regarding the relationship between smoking and cancer and mortality in the Asian population.

Because smoking is a prevalent environmental factor, we believe that studying the association between smoking and IBD is crucial at this moment when IBD incidence and prevalence are increasing, and the results can help physicians provide appropriate recommendations regarding the adjustment of smoking habits in Asian patients with either UC or CD. In this study, we aimed to define the trend and prevalence of smoking in IBD patients with a history of hospitalization and to compare the prevalence of smoking among IBD patients with previous hospitalization and Taiwan population. Furthermore, we performed the comparison between smokers versus non-smokers to discover the effect of smoking on the severity (measured as biologics usage, admission, and emergency room [ER] visits) and outcomes (measured as operation, cancer, and survival) of UC and CD.

## Results

### Prevalence of smoking between IBD patients and their matched controls

**Figure 1:**
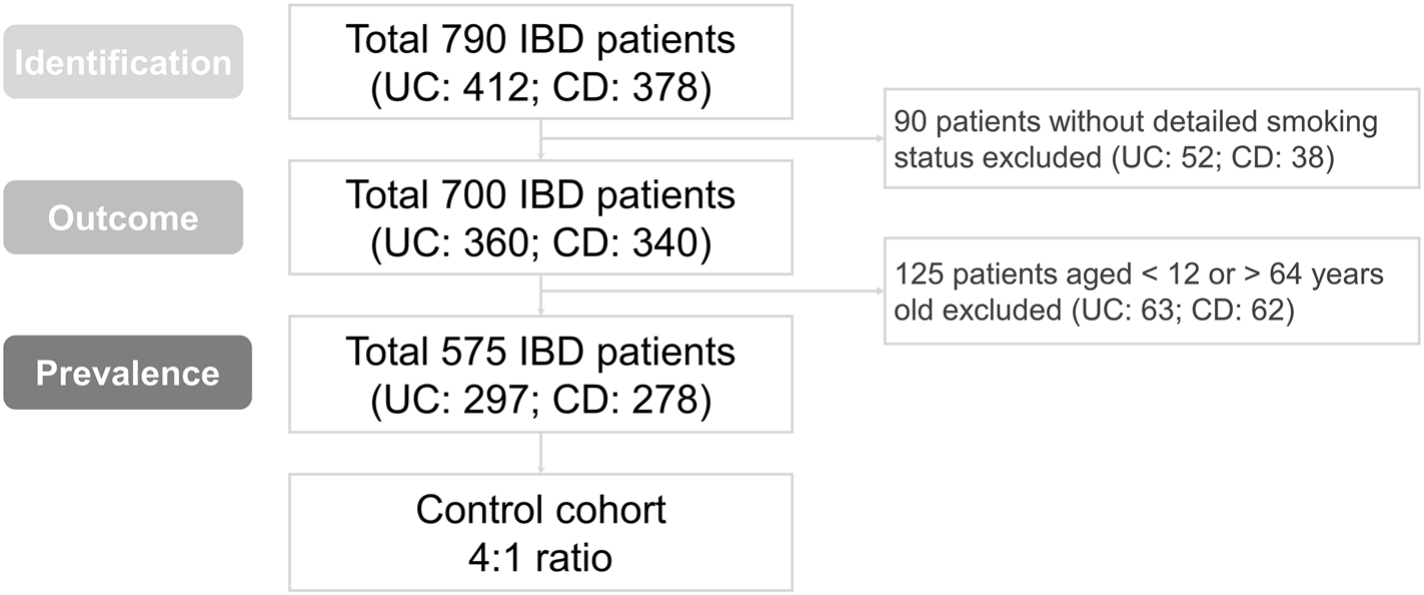
Flowchart of IBD patient selection for the study.

The flowchart of IBD patient selection for the study was shown in Fig. 1. In total, 790 patients (UC: 412 and CD: 378) were diagnosed with IBD and ever admitted during the study period. Among them, the detailed smoking history of 700 IBD patients was available. These 700 IBD patients (UC: 360 and CD: 340) were enrolled, and their data were analyzed for determining the influence of smoking on outcomes. The median follow-up periods of UC and CD patients were 104 and 85 months, respectively. We included 2 different control groups for each compared group (UC and CD), paired according to sex, age and presence of comorbidities, to compare the prevalence of smoking between IBD patients and matched controls. Only 575 patients (UC: 297 and CD: 278) with complete information of comorbidities (hypertension, diabetes, hyperlipidemia) and aged from 12 to 64 years old were enrolled with the median follow up time as 100 and 92.5 months, respectively.

As shown in Tables 1 and 2, IBD patients in Taiwan were predominantly men (UC: 61.6% and CD: 70.1%). The median age of patients at diagnosis of UC and CD was 42 and 27 years, respectively. Individual matching with controls according to age, sex, and comorbidities (hypertension, diabetes, and hyperlipidemia) at a 1:4 ratio was conducted for UC and CD cohorts. Smoking (past and current) prevalence in the matched controls of each group was 361/1188 (30.4%) and 246/1112 (22.1%), respectively. Smoking prevalence was significantly lower in UC patients than in the matched controls (20.9% vs. 30.4%, *p* < 0.01), and no difference was observed between the CD and matched control groups (19.8% vs. 22.1%, *p* = 0.60). The disease extent of patients at UC diagnosis was mainly extensive (45.5%), and the mean Modified Mayo Score was 2.2. Among CD patients, the location at diagnosis was mainly colonic (47.4%), and the behavior at diagnosis was mainly nonstricturing and nonpenetrating (65.0%). The detailed clinical information is listed in Tables 1 and 2.

**Table 1:** Clinical characteristics of patients with UC and matched controls.

	UC patients N = 297*	Controls N = 1188	*p* value
**Sex (n, %)**			
Male	183 (61.6%)	732 (61.6%)	
Female	114 (38.4%)	456 (38.4%)	
Age at diagnosis, year (median, range)	42 (12, 64)	N/A	
Follow-up, month (median, range)	100 (0, 691)	N/A	
**Comorbidities (n, %)**			
Hypertension	35 (11.8%)	140 (11.8%)	
Diabetes mellitus	11 (3.7%)	44 (3.7%)	
Dyslipidemia	22 (7.4%)	88 (7.4%)	
Viral hepatitis	49 (16.5%)	N/A	
Coronary artery disease	14 (4.7%)	N/A	
Chronic kidney disease	2 (0.7%)	N/A	
**Extent at diagnosis (n, %)**			
Proctitis	44 (17.3%)	N/A	
Left-sided	95 (37.3%)	N/A	
Extensive	116 (45.5%)	N/A	
The Modified Mayo Score (Mean, SD)	2.2 (0.8)	N/A	
**Smoking (n, %)**			< 0.01
Never	235 (79.1%)	827 (69.6%)	
Past	31 (10.4%)	117 (9.9%)	
Current	31 (10.4%)	244 (20.5%)	

*Inclusion criteria: patients aged 12–64 years with complete information on hypertension, diabetes, hyperlipidemia, and smoking status.

**Table 2:** Clinical characteristics of patients with CD and matched controls.

	CD patients N = 278*	Controls N = 1112	*p* value
**Sex (n, %)**			
Male	195 (70.1%)	780 (70.1%)	
Female	83 (29.9%)	332 (29.9%)	
Age at diagnosis, year (median, range)	27 (12, 63)	N/A	
Follow-up, month (median, range)	92.5 (0, 465)	N/A	
**Comorbidities (n, %)**			
Hypertension	22 (7.9%)	88 (7.9%)	
Diabetes mellitus	7 (2.5%)	28 (2.5%)	
Dyslipidemia	7 (2.5%)	28 (2.5%)	
Viral hepatitis	32 (11.5%)	N/A	
Coronary artery disease	7 (2.5%)	N/A	
Chronic kidney disease	9 (3.2%)	N/A	
**Location at diagnosis (n, %)**			
Terminal ileal (L1)	41 (16.7%)	N/A	
Colonic (L2)	116 (47.4%)	N/A	
Ileocolonic (L3)	88 (35.9%)	N/A	
**Behavior at diagnosis (n, %)**			
Non-stricturing & non-penetrating (B1)	160 (65.0%)	N/A	
Stricturing (B2)	65 (26.4%)	N/A	
Penetrating (B3)	21 (8.5%)	N/A	
SES-CD (Median, SD)	7 (5)	N/A	
**Smoking (n, %)**			0.60
Never	223 (80.2%)	866 (77.9%)	
Past	15 (5.4%)	77 (6.9%)	
Current	40 (14.4%)	169 (15.2%)	

*Inclusion criteria: patients aged 12–64 years with complete information on hypertension, diabetes, hyperlipidemia, and smoking status.

### Trend for prevalence of current smoking in the IBD and control groups

**Figure 2:**
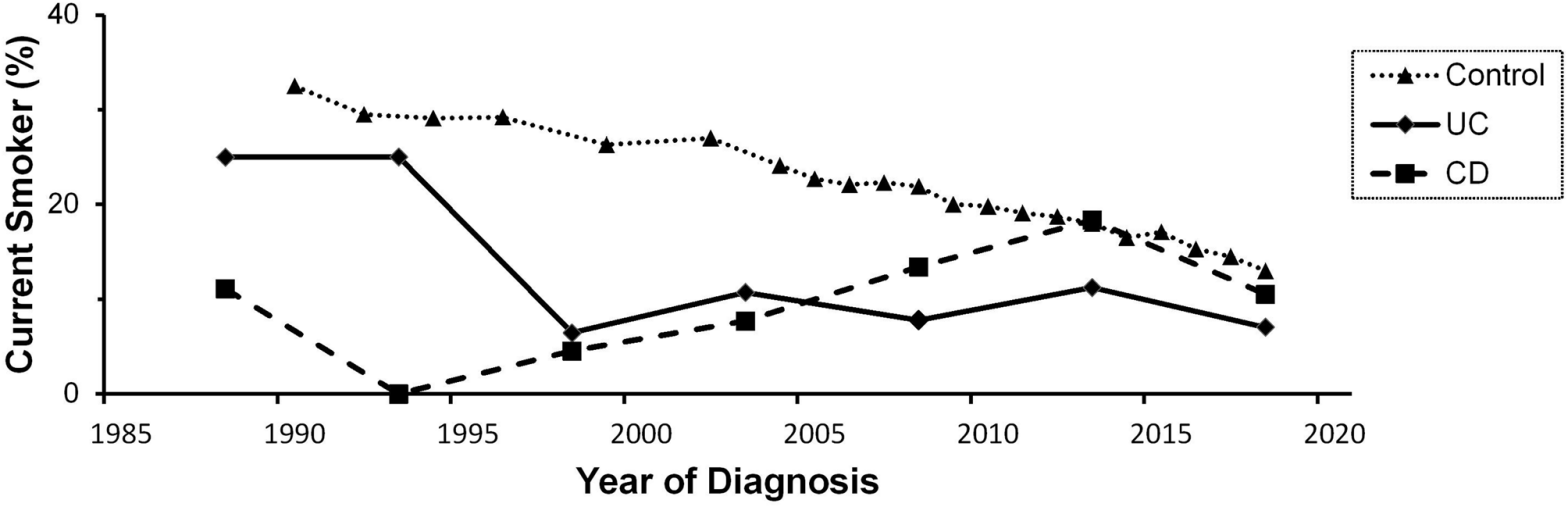
The proportion of current smokers during IBD diagnosis and control selection, stratified by year. Patients were classified based on year at diagnosis (before 1990, 1991–1995, 1996–2000, 2001–2005, 2006–2010, 2011–2015, and 2016–2020). The crude prevalence of current smoking decreased from 32.5% (1990) to 13% (2018) in the control group (*p* < 0.01) and 25% (before 1990) to 7% (2016–2020) in UC patients (*p* = 0.04), whereas the prevalence of current smoking did not decrease over time and remained stable at approximately 9% in CD patients (*p* = 0.89).

To explore the trend of chronicity change for smoking prevalence, we stratified the period as before 1990 and then every 5 years until 2020. The crude prevalence of current smoking among IBD patients at diagnosis and among controls stratified by years is shown in Fig. 2. The crude prevalence of current smoking decreased from 32.5% (1990) to 13% (2018) in the control group (p < 0.01) and 25% (before 1990) to 7% (2016–2020) in the UC group (*p* = 0.04), whereas the prevalence of current smoking did not decrease over time and remained stable at approximately 9% in CD patients (*p* = 0.89).

### Smoking and disease outcomes of UC patients

For exploring the effect of smoking on disease outcomes, 360 UC patients with a detailed smoking history were enrolled and their data analyzed. Among them, 21% (75 patients) were smokers (past and current smokers), and 79% (285 patients) were nonsmokers (Table 3). Furthermore, smokers were more often men (94.7% vs. 52.3%, *p* < 0.01) and older (48 vs. 39 years old, *p* < 0.01) than nonsmokers. However, no difference was observed in the follow-up duration, medication use, number of ER visits, disease severity, surgery rate, and laboratory data between these two groups.

**Table 3:** Disease outcomes of smokers and nonsmokers with UC.

UC patients	Smoking N = 75	Non-smoking N = 285	*p* value
Male	71 (94.7%)	149 (52.3%)	< 0.01*
Age at diagnosis, median (Q1, Q3)	48 (42, 57)	39 (26, 52)	< 0.01*
Follow-up, month (median, range)	104 (47, 180)	98 (40, 174)	0.90
**Medications**			
Steroid (n, %)	53 (70.7%)	208 (73.0%)	0.69
5-ASA (n, %)	72 (96.0%)	275 (96.5%)	0.74^†^
Azathioprine (n, %)	26 (34.7%)	119 (41.8%)	0.27
Other immunomodulator ^a^ (n, %)	5 (6.7%)	23 (8.1%)	0.69
Advanced therapy ^b^ (n, %)	23 (30.7%)	74 (26.0%)	0.41
Times of admission (Mean, SD)	1.6 (2.1)	2.5 (4.9)	0.02*
Times of ER (Mean, SD)	0.9 (1.5)	1.0 (1.7)	0.68
The Modified Mayo Score (Mean, SD)	2.3 (0.9)	2.25 (0.8)	0.96
**Surgery (n, %)**	11 (14.7%)	22 (7.7%)	0.06
Fistula, perforation, abscess (n, %)	4 (5.3%)	12 (4.2%)	0.75^†^
Stricture (obstruction) (n, %)	1 (1.3%)	3 (1.1%)	1.00^†^
Bleeding, refractory, or else (n, %)	3 (4.0%)	3 (1.1%)	0.11^†^
Malignancy (n, %)	3 (4.0%)	4 (1.4%)	0.16^†^
Cancer (n, %)	12 (16.0%)	19 (6.7%)	0.01*
**Death (n, %)**	12 (16.0%)	14 (4.9%)	< 0.01*
Infection (n, %)	5 (6.7%)	8 (2.8%)	0.16^†^
Malignancy (n, %)	4 (5.3%)	5 (1.8%)	0.09^†^
Cardiovascular disease (n, %)	2 (2.7%)	0	0.04^†^
Others (n, %)	1 (1.3%)	1 (0.4%)	0.37^†^
Baseline hemoglobin (mg/dL) (Mean, SD)	12.7 (2.5)	12.1 (2.4)	0.11
Baseline CRP (mg/dL) (Mean, SD)	2.9 (4.9)	2.2 (3.5)	0.29
Baseline albumin (mg/dL) (Mean, SD)	3.8 (0.8)	3.9 (0.7)	0.70
**Extent at diagnosis (n, %)**			0.86
Proctitis	12 (16.9%)	41 (15.8%)	
Left-sided	23 (32.4%)	93 (35.9%)	
Extensive	36 (50.7%)	125 (48.3%)	

^a^Methotrexate, tacrolimus, and cyclosporine.

^b^Anti-TNF, vedolizumab, ustekinumab, p19 antibody, and Jak1 inhibitor.

^†^Using Fisher’s exact test.

* Significant (*p* < 0.05).

Although smoking was associated with fewer hospitalizations (1.6 vs. 2.5, *p* = 0.02), smokers had a higher rate of new cancer development (16.0% vs. 6.7%, *p* = 0.01) and higher mortality (16.0% vs. 4.9%, *p* < 0.01) than nonsmokers. Various cancers types were involved among smokers with UC with a high rate of cancer, including colorectal cancer, lymphoma, prostate cancer, renal cell carcinoma, and hepatocellular carcinoma. To explore the etiology of the higher mortality rate in UC smokers than in nonsmokers, we have conducted a subgroup analysis based on the cause of death. We found that cardiovascular disease (CVD) was higher in the smoker group (2.7% vs. 0.0%, *p* = 0.04), and infection (6.7% vs. 2.8%, *p* = 0.16) and malignancy (5.3% vs. 1.8%, *p* = 0.09) was slightly increased in smokers with UC, but the findings were not statistically significant.

### Smoking prevalence and disease outcomes of CD patients

Among 340 patients with CD, 19% (63 patients) were smokers (past and current smokers), and 81% (277 patients) were nonsmokers (Table 4). Smokers were more often men (93.7% vs. 61.7%, *p* < 0.01) and older (44 vs. 22 years old, *p* < 0.01) than nonsmokers. Among patients with CD, smokers had different disease locations and behaviors compared with nonsmokers (location: *p* < 0.01 and behavior: *p* = 0.03) at diagnosis. Smokers tended to have CD located in the terminal ileum (23.6% vs. 14.3%) and ileocolon (42.3% vs. 33.2%). Smokers had high rates of stricturing (32.7% vs. 20.9%) and penetrating (12.7% vs. 6.7%) disease at diagnosis.

**Table 4:** Disease outcomes of smokers and nonsmokers with CD.

CD patients	Smoking N = 63	Non-smoking N = 277	*p* value
Male	59 (93.7%)	171 (61.7%)	< 0.01*
Age at diagnosis, median (Q1,Q3)	44 (32, 53)	22 (15, 36)	< 0.01*
Follow-up, month (median, range)	85 (34, 129)	84 (43,153)	0.13
**Medications**			
Steroid (n, %)	50 (79.4%)	231 (83.4%)	0.45
5-ASA (n, %)	55 (87.3%)	258 (93.1%)	0.12
Azathioprine (n, %)	41 (65.1%)	190 (68.6%)	0.59
Other immunomodulator ^a^ (n, %)	7 (11.1%)	34 (12.3%)	0.80
Advanced therapy ^b^ (n, %)	32 (50.8%)	133 (48.0%)	0.69
Times of admission (Mean, SD)	3.1 (2.8)	4.8 (6.1)	< 0.01*
Times of ER (Mean, SD)	1.8 (2.8)	2.3 (3.4)	0.32
SES-CD (Mean, SD)	6.5 (4.6)	7.3 (5.3)	0.30
**Surgery (n, %)**	38 (60.3%)	106 (38.3%)	< 0.01*
Fistula, perforation, abscess (n, %)	18 (28.6%)	60 (21.7%)	0.24
Stricture (obstruction) (n, %)	15 (23.8%)	34 (12.3%)	0.02*
Bleeding, refractory, or else (n, %)	8 (12.7%)	20 (7.2%)	0.15
Malignancy (n, %)	5 (7.9%)	7 (2.5%)	0.05*^†^
Others (n, %)	5 (7.9%)	6 (2.2%)	0.03*^†^
Cancer (n, %)	4 (6.4%)	14 (5.1%)	0.75^†^
Death (n, %)	2 (3.2%)	10 (3.6%)	1.00^†^
Baseline hemoglobin (mg/dL) (Mean, SD)	12.4 (2.3)	11.6 (2.2)	0.02*
Baseline CRP (mg/dL) (Mean, SD)	3.7 (5.7)	3.8 (4.8)	0.89
Baseline albumin (mg/dL) (Mean, SD)	3.7 (0.8)	3.8 (0.8)	0.25
**Location at diagnosis (n, %)**			< 0.01*
Terminal ileal (L1)	13 (23.6%)	34 (14.3%)	
Colonic (L2)	16 (29.1%)	125 (52.5%)	
Ileocolonic (L3)	26 (47.3%)	79 (33.2%)	
**Behavior at diagnosis (n, %)**			0.03*
Non-stricturing and non-penetrating (B1)	30 (54.6%)	173 (72.4%)	
Stricturing (B2)	18 (32.7%)	50 (20.9%)	
Penetrating (B3)	7 (12.7%)	16 (6.7%)	

^a^Methotrexate, tacrolimus, and cyclosporine.

^b^Anti-TNF, vedolizumab, ustekinumab, p19 antibody, and Jak1 inhibitor.

^†^Using Fisher’s exact test.

*Significant (*p* < 0.05).

Smokers had fewer hospitalizations (3.1 vs. 4.8, *p* < 0.01) but an increased risk of CD-related surgery (60.3% vs. 38.3%, *p* < 0.01) and increased baseline hemoglobin (12.4 vs. 11.6, *p* = 0.02) than nonsmokers. No difference was noted in the follow-up duration, medication use, number of ER visits, disease severity, mortality, and baseline CRP and albumin level between these two groups.

On the basis of a subgroup analysis for surgical indication, smokers had an increased surgery risk due to stricture or obstruction of the bowel (23.8% vs. 12.3%, *p* = 0.02). Surgery rates due to other etiologies, such as fistula, perforation, bleeding, or malignancy, were not significantly different between the two groups.

### Effect of smoking cessation before IBD diagnosis

We explored the effect of smoking cessation and compared the differences in outcomes between past smokers and current smokers at diagnosis. Among 75 smokers with UC, 51% (38 patients) were past smokers, and 49% (37 patients) were current smokers. Among 63 smokers with CD, 30% (19 patients) were past smokers, and 70% (44 patients) were current smokers. When comparing the disease phenotypes and outcomes, we did not find any statistical difference between past and current smokers, although high proportions of current and past smokers with CD had strictures and inflammatory disease phenotypes, respectively (Supplementary tables 1 and 2). The small sample size and the follow-up period (median as 90–100 months) < 20 years might explain these results.

## Discussion

To the best of our knowledge, this is the largest study to explore the effects of smoking on Asian IBD patients. In population-based and hospital-based cohorts, smoking prevalence was approximately 20% in UC patients at diagnosis and 30% in matched controls. Patients with UC had significantly lower smoking prevalence than controls. In the past decades, the crude prevalence of smoking in UC patients decreased gradually, but the incidence and prevalence of UC increased in Taiwan^13^. Furthermore, our results showed that UC smokers had a low hospitalization risk. Thus, smoking is a protective factor for UC in the Asian population. This result was similar to that of the Western population^3,5,6^.

For CD, our results demonstrated that smoking prevalence was not significantly different between patients with CD at diagnosis and matched controls. The smoking prevalence in Western countries is approximately 31–78%^1,5,14–17^, whereas in our study, the smoking prevalence (past and current smoking) was only 19.8% for CD patients and 22.1% for controls. Thus, smoking is not a risk factor for CD in the Asian population, which is different from the situation reported in the Western population^4,5^. In this study, controls were matched with IBD patients by factors including age, sex, and comorbidities. As the UC patients were older than CD patients (diagnosed age: 42 vs 27 years-old), the UC matched control group was older than CD mated control group. The prevalence of current smoking decreased over time in the control group, therefore the smoking prevalence of UC mated control group was higher than CD matched control group.

We also analyzed the trend of smoking prevalence in IBD patients and controls by the time of diagnosis. The smoking prevalence in Taiwan IBD patients is in accordance to the results from WHO, with the age-standardized prevalence of tobacco smoking in 2016 for both sexes globally being 19.9%, lowest at 9.8% in Africa, highest at 29.4% in Europe, and intermediate at 16.9% in South East Asia^2^. We found that the proportion of current smokers decreased in controls and UC patients but fluctuated without specific changes in CD patients over time.

Although smoking might prevent UC, our results showed that smoking was correlated with more new cancers and a high mortality rate. It included various cancer types, including colorectal cancer, lymphoma, prostate cancer, renal cell carcinoma, and hepatocellular carcinoma, but was not limited to cancers of gastrointestinal tract. The result may be attributed to the carcinogenic effect of smoking. Tobacco smoking is a well-established risk factor for several cancers and is the single largest cause of cancer worldwide. Furthermore, smoking is classified as a group 1 carcinogen by the International Agency for Research on Cancer^18^. We also found that compared with UC nonsmokers, UC smokers had a higher mortality risk. In addition to cancer, CVD risk increased in UC smokers. This is not too surprising because smoking is a well-known risk factor for CVD and is responsible for 20% of CVD deaths^19^.

Our results showed that for CD patients, smokers tended to have more ileal involvement, but nonsmokers tended to have more colonic involvement. For CD disease behavior, smokers tended to have more stricturing (32.73% vs. 20.92%) and penetrating disease (12.73% vs. 6.69%), but nonsmokers tended to have more inflammatory type. These results are consistent with previous reports^9,20^. CD smokers had a higher surgical rate than nonsmokers in the current study. We performed subgroup analysis by dividing the surgery indication into five major categories: penetrating disease, stricture-related obstruction, bleeding, malignancy, and others. In our study, stricture and penetrating diseases accounted for 86.7% (33/38) of the indication for surgery. Furthermore, a similar result was reported by Louis^7^. Stricturing and penetrating diseases have been reported to contribute to a high surgery rate^21,22^. Moreover, a Korean retrospective multicenter cohort study that enrolled 728 CD patients demonstrated that stricturing, penetrating disease behavior, and smoking habits at the time of diagnosis are independent predictors of CD-related surgery^23^. Cosnes reported that surgery risk increased in patients who smoked and did not undergo immunosuppressive therapy^24^. However, increased use of immunosuppressive or biologic therapy was not observed among smokers compared with nonsmokers. Therefore, the compensating effect of immunosuppressants and biologic agents for the influence of smoking cannot be observed in our studies. Smoking had no significant detrimental effect on medical requirements in Indian CD patients was also reported in Arora’s study^25^.

We compared disease outcomes of current smokers and past smokers to evaluate the beneficial effect of smoking cessation noted in previous studies^26,27^. However, we failed to identify differences in disease outcomes between these two groups because the sample size of ex-smokers was small. Additionally, this might result from the short period of smoking cessation because studies^4^ have shown that the effect of smoking may persist for 20 years.

Our study has some limitations. First, given the integrity of medical records, we only included patients who were hospitalized at least once. Disease severity in this study cohort may have been overestimated. However, both smoking and nonsmoking IBD patients were enrolled using the same criteria; hence, the result should not be affected too much. Second, the smoking status of each patient was recorded at different time points based on the time of diagnosis. By contrast, the smoking status of each control was recorded in the same year. This may cause bias in our study because smoking prevalence may change over time. Third, this was a retrospective study.

Nonetheless, the strength of this study is that we used individuals from a nationwide health survey as matched controls. Additionally, the number of patients diagnosed with IBD from 2001 to 2015 in Taiwan was about 3800^28^, the substantial case number of this study makes our study population representative of the IBD population of Taiwan. We performed a comprehensive review to analyze the effect of smoking on IBD by comparing various outcome types between smokers and nonsmokers.

## Conclusion

Based on our study with Taiwan IBD patients with a history of hospitalization, smoking is a protective factor for UC occurrence and is associated with a low hospitalization rate, but it has no effect on CD occurrence. Smokers tended to have more extensive involvement and aggressive behavior of CD, resulting in an increased surgical rate. Smoking increased the risk of cancer and mortality in UC patients. For patients diagnosed with IBD, our results provide evidence that smoking cessation decreases the risks of cancer and mortality in UC patients and decreases complications and surgery in CD patients.

## Methods

### Patients

This hospital-based cohort study was conducted through a chart review of the smoking status and clinical characteristics of IBD patients at the National Taiwan University Hospital (NTUH). Patients diagnosed with IBD from January 1, 1963, to May 25, 2020 and had been hospitalization at least once in NTUH were enrolled. This study was approved by the Research Ethics Committee Office of National Taiwan University Hospital (ID of the approval: 202003078RINC). All methods were carried out in accordance with relevant guidelines and regulations. The Institutional Review Board of NTUH allowed to waive the informed consent because of the retrospective nature of the study and the analysis used anonymous clinical data. CD and UC were diagnosed by gastroenterologists based on clinical manifestations, endoscopic findings, and pathological findings. The smoking status of each patient was recorded at first diagnosis of IBD. Patients without a complete record of smoking status were excluded. The follow-up duration was defined from the time of first diagnosis of either UC or CD, to the time of most recent outpatient visit, as of time of inclusion in this study.

### Smoking status definition

The smoking status of each patient was retrieved from the medical record. We defined nonsmokers as those who had never smoked or smoked less than 100 cigarettes in their lifetime, past smokers as those who had smoked at least 100 cigarettes in their lifetime but had quit smoking at the time of interview, and current smokers as those who had smoked 100 cigarettes in their lifetime and currently still smoke cigarettes at the time of diagnosis.

### Study design

To investigate the effect of smoking on IBD, non-IBD controls were selected from the database from the National Health Interview Survey conducted in 2009 by the Health Promotion Administration of Taiwan and were matched with IBD patients at a ratio of 4:1. Controls were matched by factors including age, sex, and comorbidities (hypertension, diabetes, and hyperlipidemia). Only patients aged 12–64 years were included to fit with the age range of participants in the National Health Interview Survey. After matching, percentages of nonsmokers, past smokers, and current smokers in both IBD and control groups were analyzed.

For determining the trend of smoking prevalence among IBD patients, we analyzed the proportion of current smokers among IBD patients and controls in different periods. Patients were categorized based on diagnosis year (before 1990, 1991–1995, 1996–2000, 2001–2005, 2006–2010, 2011–2015, and 2016–2020). Control data were obtained from the survey of Taiwan Tobacco & Liquor Corporation (1990–1996), National Survey of Smoking Prevalence and Behaviors of Adults in Taiwan (1999), National Survey on Knowledge, Attitude, Practice of Health Promotion (2002), and Adult Smoking Behavior Surveillance System (2004–2018). These data were compiled by the government of Taiwan and published as an open access database^29^.

To examine the effect of smoking on the outcomes of IBD, clinical characteristics including disease phenotype, medication use, number of admissions, number of ER visits, surgery, new cancer development, survival status, endoscopic severity, baseline hemoglobin, c-reactive protein (CRP), and albumin were retrieved and analyzed. Outcomes of both smoking (including past and current smokers) and nonsmoking groups were analyzed. Furthermore, we compared the outcomes of current smokers and past smokers to evaluate the effect of smoking cessation.

### Clinical outcomes

We analyzed clinical outcomes by examining characteristics, including disease phenotype, medication use, number of admissions, number of ER visits, surgery, new cancer development, survival status, endoscopic severity, baseline hemoglobin, CRP, and albumin. The phenotype of IBD, including the extent (proctitis, left sided, and extensive) of UC and the location (ileal, colonic, and ileocolonic) and behavior (nonstricturing and nonpenetrating, stricturing, and penetrating) of CD, was defined according to Montreal classification. Information on medication use, including steroid, 5-ASA (5-aminosalicylic acid), azathioprine, other immunomodulators (methotrexate, tacrolimus, and cyclosporine), and advanced therapy (including anti-TNF (tumor necrosis factor), vedolizumab, ustekinumab, p19 antibody, and JAK (Janus kinase) inhibitors), was retrieved and analyzed. Admissions and ER visits only due to acute exacerbation were included, and admissions due to scheduled surgery, biological treatment, or other examinations were excluded. Surgery was defined as IBD-related surgeries such as bowel resection, fistulectomy, and balloon dilatation. Surgeries were further divided into subgroups based on the indications of each surgery. Furthermore, deaths were divided into subgroups according to the cause. Cancers that developed after but not before IBD diagnosis were analyzed. The initial endoscopy report in NTUH was recorded. Endoscopic severity was measured based on the Modified Mayo Score (normal [0], mild [1], moderate [2], and severe [3]) for UC patients and the Simple Endoscopic Score for Crohn Disease (SES-CD) for CD patients.

### Statistical analysis

Statistical analyses were performed using SAS (Statistical Analysis System) 9.4, SAS Institute. Age at diagnosis and follow-up duration are presented as median and interquartile range. Other numerical data are presented as mean and standard deviation (SD). Categorial data are presented as percentages. Differences between groups were analyzed using the *t* test for numerical data and the chi-square test and Fisher’s exact test for categorial data. The *p* values of < 0.05 were considered significant.

## Ethics declarations

Research Ethics Committee Office, National Taiwan University Hospital ID of the approval: 202003078RINC.

## Supplementary Information

Supplementary Information.

## Data Availability

The anonymous patient data are available in the database of National Taiwan University Hospital (NTUH) and will be shared on reasonable request to the corresponding author. The data of matched control are available in National Health Interview Survey conducted by National Health Research Institutes (NHRI) of Taiwan and will be shared on reasonable request to the NHRI. The data of smoking prevalence of Taiwan are available in https://www.hpa.gov.tw/Pages/Detail.aspx?nodeid=1718&pid=9913 (in Chinese).

## References

[1] Niewiadomski O (2015). Prospective population-based cohort of inflammatory bowel disease in the biologics era: Disease course and predictors of severity. J. Gastroenterol. Hepatol..

[2] World Health Statistics data visualizations dashboard http://apps.who.int/gho/data/node.sdg.3-a-viz?lang=en.

[3] Thomas T (2019). Global smoking trends in inflammatory bowel disease: A systematic review of inception cohorts. PLoS ONE.

[4] Harries AD, Baird A, Rhodes J (1982). Non-smoking: a feature of ulcerative colitis. Br. Med. J..

[5] Mahid SS, Minor KS, Soto RE, Hornung CA, Galandiuk S (2006). Smoking and inflammatory bowel disease: a meta-analysis. Mayo Clin. Proc..

[6] Higuchi LM (2012). A prospective study of cigarette smoking and the risk of inflammatory bowel disease in women. Am. J. Gastroenterol..

[7] Louis E (2003). Early development of stricturing or penetrating pattern in Crohn's disease is influenced by disease location, number of flares, and smoking but not by NOD2/CARD15 genotype. Gut.

[8] Joyce MR, Hannaway CD, Strong SA, Fazio VW, Kiran RP (2013). Impact of smoking on disease phenotype and postoperative outcomes for Crohn's disease patients undergoing surgery. Langenbecks Arch. Surg..

[9] Aldhous MC (2007). Does cigarette smoking influence the phenotype of Crohn's disease? Analysis using the Montreal classification. Am. J. Gastroenterol..

[10] Takahashi H (2014). Second peak in the distribution of age at onset of ulcerative colitis in relation to smoking cessation. J. Gastroenterol. Hepatol..

[11] Wang P (2018). Smoking and Inflammatory Bowel Disease: A Comparison of China, India, and the USA. Dig. Dis. Sci..

[12] Zhai H (2017). Current smoking improves ulcerative colitis patients' disease behaviour in the northwest of China. Prz Gastroenterol..

[13] Yen HH (2019). Epidemiological trend in inflammatory bowel disease in Taiwan from 2001 to 2015: a nationwide populationbased study. Intest. Res..

[14] Lakatos PL (2013). Is current smoking still an important environmental factor in inflammatory bowel diseases? Results from a population-based incident cohort. Inflamm Bowel Dis..

[15] Chhaya V (2016). Emerging trends and risk factors for perianal surgery in Crohn's disease: a 20-year national population-based cohort study. Eur. J. Gastroenterol. Hepatol..

[16] Romberg-Camps MJ (2009). Inflammatory Bowel Disease in South Limburg (the Netherlands) 1991–2002: Incidence, diagnostic delay, and seasonal variations in onset of symptoms. J. Crohns Colitis.

[17] Ott C (2008). The incidence of inflammatory bowel disease in a rural region of Southern Germany: a prospective population-based study. Eur. J. Gastroenterol. Hepatol..

[18] Secretan B (2009). A review of human carcinogens—Part E: tobacco, areca nut, alcohol, coal smoke, and salted fish. Lancet Oncol..

[19] Cole TB (2019). Smoking cessation and reduction of cardiovascular disease risk. JAMA.

[20] Russel MG (1998). Inflammatory bowel disease: is there any relation between smoking status and disease presentation? European Collaborative IBD Study Group. Inflamm Bowel Dis..

[21] Chan WPW, Mourad F, Leong RW (2018). Crohn's disease associated strictures. J. Gastroenterol. Hepatol..

[22] Li Y (2019). Current diagnosis and management of Crohn's disease in China: results from a multicenter prospective disease registry. BMC Gastroenterol..

[23] Moon CM (2014). Clinical features and predictors of clinical outcomes in Korean patients with Crohn's disease: a Korean association for the study of intestinal diseases multicenter study. J. Gastroenterol. Hepatol..

[24] Cosnes J, Carbonnel F, Beaugerie L, Le Quintrec Y, Gendre JP (1996). Effects of cigarette smoking on the long-term course of Crohn's disease. Gastroenterology.

[25] Arora U (2018). Effect of oral tobacco use and smoking on outcomes of Crohn's disease in India. J. Gastroenterol. Hepatol..

[26] Cosnes J (1999). Effects of current and former cigarette smoking on the clinical course of Crohn's disease. Aliment Pharmacol. Ther..

[27] Collaborators GBDT (2017). Smoking prevalence and attributable disease burden in 195 countries and territories, 1990–2015: a systematic analysis from the Global Burden of Disease Study 2015. Lancet.

[28] Lin WC (2019). Trends and risk factors of mortality analysis in patients with inflammatory bowel disease: a Taiwanese nationwide population-based study. J. Transl. Med..

[29] Health Promotion Administration, Ministr of Health and Welfare: Adult Smoking Behavior Surveillance System. www.hpa.gov.tw/Pages/Detail.aspx?nodeid=1718&pid=9913.

