# Supplementary material for: Effect of smoking on the development and outcomes of inflammatory bowel disease in Taiwan: a hospital-based cohort study
Source: Sci Rep. 2022 May 10;12:7665. doi: 10.1038/s41598-022-11860-y (PMC9090732; doi:10.1038/s41598-022-11860-y)
Supplement: Supplementary file 1 — Supplementary Information. [file 41598_2022_11860_MOESM1_ESM.docx]

**Supplementary Table 1 -** Disease outcomes of past smokers and current smokers with UC

| UC patients | Past smoker  N = 38 | Current smoker  N = 37 | *p* value |
| --- | --- | --- | --- |
| Medications |  |  |  |
| Steroid use (n, %) | 25 (65.8%) | 28 (75.7%) | 0.35 |
| 5-ASA use (n, %) | 37 (97.4%) | 35 (94.6%) | 0.61 |
| Azathioprine use (n, %) | 12 (31.6%) | 14 (37.8%) | 0.57 |
| Other immunomodulator use ^†^ (n, %) | 2 (5.3%) | 3 (8.1%) | 0.67 |
| Advanced therapy ^‡^ (n, %) | 12 (31.6%) | 11 (29.7%) | 0.86 |
| Times of admission (Mean, SD) | 1.4 (1.6) | 1.7 (2.5) | 0.56 |
| Times of ER (Mean, SD) | 0.8 (1.5) | 1.0 (1.5) | 0.55 |
| The Modified Mayo Score (Mean, SD) | 2.2 (0.8) | 2.3 (0.9) | 0.78 |
| Surgery (n, %) | 7 (18.4%) | 4 (10.8%) | 0.35 |
| Cancer (n, %) | 5 (13.2%) | 7 (18.9%) | 0.50 |
| Death (n, %) | 7 (18.4%) | 5 (13.5%) | 0.56 |
| Baseline hemoglobin (mg/dL) (Mean, SD) | 12.9 (2.5) | 12.4 (2.6) | 0.44 |
| Baseline CRP (mg/dL) (Mean, SD) | 3.1 (5.9) | 2.7 (3.7) | 0.70 |
| Baseline albumin (mg/dL) (Mean, SD) | 3.8 (0.8) | 3.8 (0.8) | 0.88 |
| Extent at diagnosis (n, %) |  |  | 0.35 |
| Proctitis | 6 (16.6%) | 6 (17.1%) |  |
| Left-sided | 9 (25.0%) | 14 (40.0%) |  |
| Extensive | 21 (58.3%) | 15 (42.9%) |  |

^†^Methotrexate, tacrolimus, and cyclosporine.

^‡^Anti-TNF, vedolizumab, ustekinumab, p19 antibody, and Jak1 inhibitor.

**Supplementary Table 2 -** Disease outcomes of past smokers and current smokers with CD

| CD patients | Past smoker  N = 19 | Current smoker  N = 44 | *p* value |
| --- | --- | --- | --- |
| Medications |  |  |  |
| Steroid use (n, %) | 14 (73.7%) | 36 (81.8%) | 0.51 |
| 5-ASA use (n, %) | 16 (84.2%) | 39 (88.6%) | 0.69 |
| Azathioprine use (n, %) | 13 (68.4%) | 28 (63.6%) | 0.71 |
| Other immunomodulator use ^†^ (n, %) | 3 (15.8%) | 4 (9.1%) | 0.42 |
| Advanced therapy ^‡^ (n, %) | 11 (57.9%) | 21 (47.7%) | 0.46 |
| Times of admission (Mean, SD) | 2.9 (3.3) | 3.11 (2.6) | 0.78 |
| Times of ER (Mean, SD) | 1.5 (2.2) | 1.93 (3.1) | 0.60 |
| SES-CD (Mean, SD) | 6 (4.2) | 6.75 (4.7) | 0.58 |
| Surgery (n, %) | 10 (52.6%) | 28 (63.6%) | 0.41 |
| Cancer (n, %) | 2 (10.5%) | 2 (4.6%) | 0.58 |
| Death (n, %) | 2 (10.5%) | 0 (0.0%) | 0.09 |
| Baseline hemoglobin (mg/dL) (Mean, SD) | 11.8 (1.6) | 12.8 (2.5) | 0.13 |
| Baseline CRP (mg/dL) (Mean, SD) | 3.4 (3.9) | 3.9 (6.6) | 0.75 |
| Baseline albumin (mg/dL) (Mean, SD) | 3.7 (0.5) | 3.7 (0.9) | 0.86 |
| Location at diagnosis (n, %) |  |  | 0.55 |
| L1 | 4 (25.0%) | 9 (23.1%) |  |
| L2 | 3 (18.8%) | 13 (33.3%) |  |
| L3 | 9 (56.3%) | 17 (43.6%) |  |
| Behavior at diagnosis (n, %) |  |  | 0.10 |
| B1 | 12 (75.0%) | 18 (46.2%) |  |
| B2 | 2 (12.5%) | 16 (41.0%) |  |
| B3 | 2 (12.5%) | 5 (12.8%) |  |

^†^Methotrexate, tacrolimus, and cyclosporine.

^‡^Anti-TNF, vedolizumab, ustekinumab, p19 antibody, and Jak1 inhibitor.
